# Upon Section Plates

**Published:** 1884-12

**Authors:** D. Genese


ARTICLE VII.

### UPON SECTION PLATES.

BY D. GENESE, D. D. S.

The models I now show are those of a patient advanced in years, with a great objection to having the remaining natural teeth extracted, as he states was done with the upper case, and gave him great trouble on account of not being able to get a case to keep in its place, though he had had six plates before I saw him.

Two causes existed for the failures that have occurred.

Firstly, the irregular surface that the upper case had to be adapted to.

Secondly, the liability of the tongue (which was above the average size) forming a complete vacuum and drawing the case down even in sucking.

Having met with only two cases of this description some years ago, and at long intervals the matter passed without attention until this case came to hand, when I determined to establish the same relation between the inside of the plate and the palate, as existed between the tongue and the under surface of the plate, that was to fit it loosely and with a polished surface.

To accomplish this I made a die and counter and formed a plate of tin, I now show you this. I sealed it to the model with Fibrin varnish and burnished it down. It brought the case out fitting loosely on to this model, but the success was complete to the patient and to myself satisfactory.

The molars were also adapted to the lower case without extraction and work well.

Since the success of this particular case, I have made all my vulcanite work upon the same plan, and find suction chambers can be dispensed with, and the cases more easily kept clean, which is a great benefit as the food does not lodge in the rough places as it does upon plaster baked pieces, and the tissues are less irritated in consequence.

